# Low level maternal smoking and infant birthweight reduction: genetic contributions of *GSTT1* and *GSTM1* polymorphisms

**DOI:** 10.1186/1471-2393-12-161

**Published:** 2012-12-26

**Authors:** Asta Danileviciute, Regina Grazuleviciene, Algimantas Paulauskas, Ruta Nadisauskiene, Mark J Nieuwenhuijsen

**Affiliations:** 1 Department of Environmental Sciences, Vytautas Magnus University, Donelaicio st. 58, 44248, Kaunas, Lithuania; 2 Clinic of Obstetrics and Gynecology, Lithuanian University of Health Science, Kaunas, Lithuania; 3 Center for Research in Environmental Epidemiology (CREAL), Parc de Recerca Biomedica de Barcelona – PRBB, Barcelona, Spain

**Keywords:** Birthweight, GST polymorphisms, Smoking, Interaction

## Abstract

**Background:**

Genetic susceptibility to tobacco smoke might modify the effect of smoking on pregnancy outcomes.

**Methods:**

We conducted a case–control study of 543 women who delivered singleton live births in Kaunas (Lithuania), examining the association between low-level tobacco smoke exposure (mean: 4.8 cigarettes/day) during pregnancy, *GSTT1* and *GSTM1* polymorphisms and birthweight of the infant. Multiple linear-regression analysis was performed adjusting for gestational age, maternal education, family status, body mass index, blood pressure, and parity. Subsequently, we tested for the interaction effect of maternal smoking, *GSTT1* and *GSTM1* genes polymorphisms with birthweight by adding all the product terms in the regression models.

**Results:**

The findings suggested a birthweight reduction among light-smoking with the *GSTT1–null* genotype (−162.9 g, *P* = 0.041) and those with the *GSTM1–null* genotype (−118.7 g, *P* = 0.069). When a combination of these genotypes was considered, birthweight was significantly lower for infants of smoking women the carriers of the double-null genotypes (−311.2 g, *P* = 0.008). The interaction effect of maternal smoking, *GSTM1* and *GSTT1* genotypes was marginally significant on birthweight (−234.5 g, *P* = 0.078). Among non-smokers, genotype did not independently confer an adverse effect on infant birthweight.

**Conclusions:**

The study shows the *GSTT1–null* genotype, either presents only one or both with *GSTM1–null* genotype in a single subject, have a modifying effect on birthweight among smoking women even though their smoking is low level. Our data also indicate that identification of the group of susceptible subjects should be based on both environmental exposure and gene polymorphism. Findings of this study add additional evidence on the interplay among two key GST genes and maternal smoking on birth weight of newborns.

## Background

Environmental factors contributing to reduced birthweight are a great concern because of the well-known relation of birthweight to infant mortality and adverse health effects in later life. Recent epidemiological studies have linked maternal tobacco-smoking and other environmental exposures to increased risk of low birth weight, preterm delivery, congenital anomalies, pregnancy loss, foetal growth, birthweight [1,2]. Investigators, who have examined the issue, showed dose–response gradients in relation to the number of cigarettes smoked [3] and association between maternal smoking during pregnancy, impaired foetal growth and overweight in childhood [4]. Some studies reported an increased risks of low birth weight (LBW) and small for gestational age with heavier maternal smoking (> 10 cigarettes/day), as well as noting an increased risk for "very preterm" birth (< 35 weeks) [3]. It was reported that smoking even 20 cigarettes/day was not related to risk of preterm birth overall, but cotinine measured at the time of delivery was. A clear association and dose–response gradient was present for risk of foetal growth restriction [5] and birthweight reduction [6] and this effect was evident even in cases of a "minimal" consumption of 1–5 cigarettes per day. It was estimated that approximately 30% of growth-restricted neonates could be independently associated with maternal smoking [7].

Low birth weight of infant is recognised to be associated with demographic and environmental factors; however, tobacco-smoking remains the most important modifiable risk factor for foetal-growth restriction [8]. Maternal smoking during pregnancy, for heavy smokers, results in an approximately 150- to 245-g reduction in the average birthweight of infants [3,9,10], however, it is generally accepted that there is no safe level of exposure to cigarette smoke [11]. In infants birthweight decreased as the mother’s smoking increased, but the differences comparing 1–5 cig/day vs. 6–10 cig/day were not significant [6]. One of the explanations of tobacco smoking influence on infants birthweight is that individual genetic susceptibility to tobacco smoke might also have an effect on the foetal development [12]; – moreover, this variability may be related to the interplay of environmental exposures, such as exposure to cigarette smoke, and host-modified metabolic processes [13].

Several recent studies have represented the investigations how genetic susceptibility modulates risk of birthweight, infant birth size, small-for-gestational-age from environmental exposures such as cigarette smoke [13-16].

Numerous chemical compounds such as polycyclic aromatic hydrocarbons (PHA) in tobacco smoke are activated and detoxified by xenobiotic-metabolizing enzymes, such as glutathione S-transferase (GST) complex. Glutathione S-transferase M1 (GSTM1) is involved in the detoxification of a large number of xenobiotics [17,18]. The genetic polymorphism of *GSTM1* may be a factor in determining the individual’s susceptibility to the toxic effects of various xenobiotics, tobacco smoke being one among them. The deficiency of *GSTM1* has been shown to impaired enzyme activities, increase DNA-adduct formation and cytogenic damage [14,19], and increase the susceptibility to both tobacco-smoke xenobiotics and small-for-gestational-age [16,20]. *GSTM1* gene polymorphism is found to be present in 40 to 60% of most populations [17].

*GSTT1*-encoded enzymes catalyses the detoxification of xenobiotics, it is probable that GSTT1 plays a protective role against cell damage. The *GSTT1*-encoded enzymes are involved in the metabolism and detoxification of PAHs. It is possible that GST induction represents part of an adaptive response mechanism to chemical stress [20]. The frequency of the *GSTT1–null* allele has been reported to be 30 to 40% in Germany [21], whereas in a Swedish population, it is only 10% [22].

*GSTM1* and *GSTT1* null genes are the result of two homozygous deletions that result in a loss of functional activity [23]. Studies of the interaction between genes and toxic chemical exposure indicate that the *GSTM1–null* and *GSTT1–null* genotypes have been associated with the effect of maternal smoking on duration of gestation, birth weight, and size [13,14,16]. Several recent studies have reported that genetic susceptibility modulates the risk of adverse pregnancy outcomes from tobacco smoke [15,16,24-31].

Different results have been presented by several authors [16]. In a case–control study, controlling for several confounding factors, the authors have shown that the maternal *GSTT1–null* genotype had a 1.6-fold reduced risk of infant-growth restriction. However, after adjustment for maternal smoking (categories less than 10 cigarettes per day and more than 10 cigarettes per day), the results are not statistically significant. There is evidence that the effect of cigarette-smoke exposure depends on the population characteristics: among the Japanese, the *GSTM1–null* genotype decreases foetal growth but this effect is not observed in Caucasians [3,14].

Tobacco smoke is a complex mixture of numerous substances that include polycyclic aromatic hydrocarbons (PAHs) and N-nitrosamines among them. Recent studies have shown that there are significant associations between exposure to PAHs and reduced foetal growth and preterm birth [1]. Increasing PAH levels during pregnancy may increase the risk of foetal growth restriction [32], lung- function reduction in children, particularly for those whose mothers possessed the polymorphic CYP1A1*2A and GSTM1 deletion [26]. However, there is inconsistency in the relationship between *GSTT1* and *GSTM1* polymorphisms and smoking effects on foetal development. Variations in the *CYP1A1* and *GSTM1* genes that encode these enzymes could affect smoking behaviour by altering the levels and duration of tobacco-related PAHs and their metabolites in the body [33].

Although separate *GSTM1* and *GSTT1* gene deletions are well-studied functional variants, so far, there are only a limited numbers of studies published on the impacts of light cigarette smoking, GST metabolic gene polymorphism, and infant birth weight data.

In this study, we used a case–control design to examine the relationship between maternal smoking, GSTM1, GSTT1 polymorphism, and birth weight. We hypothesized those women with the *GSTM1*– and *GSTT1– null* genotype who are exposed to cigarette smoke during pregnancy are at elevated risk for newborn birthweight reduction.

## Methods

### Participant and outcome assessment

A prospective cohort study of pregnant women was conducted between 2007 and 2008 in Kaunas, Lithuania (Kaunas HiWATE cohort study). On their first visit to a general practitioner, all pregnant women living in Kaunas were invited to join the cohort and answer to the first questionnaire. We recruited these women for the prospective cohort study, enrolling them at 23–35 weeks of gestation at the four prenatal care clinics affiliated to the hospitals of the Kaunas University of Medicine. Participation was on a voluntary basis and the women were enrolled in the study only if they consented to participate in the cohort. The research protocol was approved by the Lithuanian Bioethics Committee and informed consent was obtained from all subjects. A special questionnaire was evolved to interview the women who agreed to participate in the genetic study and blood samples for genetic analysis was collected. Details of the methods and study subjects have been published elsewhere [34]. The subjects of this case–control study were 543 women, who delivered singleton live births at the four hospitals affiliated to the Lithuanian University of Health Science. Multiple births or newborns with major births defects were excluded.

Pregnant women of the cohort were asked to answer second questionnaire provided to them at the clinic before delivery. The interview contained a number of variables including demographics (age, education and family status); reproductive history (miscarriage); job characteristics; self-reported psychosocial stress; health behaviour; diseases; maternal smoking; paternal smoking. The self-reported stress of the respondents was assessed by the following thesis: “My daily activities are very trying and stressful”. Four respondent options were used to define stress: this describes my state (1) very well, (2) fairly well, (3) not very well, (4) not at all. Values 1 and 2 were considered to represent stress; 3 and 4 represented no stress.

The women were followed up with regard to pregnancy outcomes by the research staff. Pregnancy outcomes were ascertained chiefly from computerised hospital admission files and by abstraction of medical records. In this study, infant birthweight was measured in the delivery room by a trained nurse and was accurate to 1 g. The age of gestation was calculated using the data of birth as reported on the birth certificate and the 1st day of the last menstrual period as was ascertained at first interview, and by ultrasound examination.

### Smoking exposure

Data regarding smoking behaviour were acquired through face-to-face interviewing. The trained research assistant in person in the hospital setting asked the women to report their daily cigarette consumption both before and during pregnancy. Woman had to answer the questions, “How many cigarettes did you smoke before pregnancy?” and “How many cigarettes did you smoke during pregnancy?” A mother was defined as smokers if she reported smoking at least one cigarette per day during pregnancy. In this study, data on mothers were categorised into two groups with respect to their cigarette smoking habits: those who did not smoke and those women who continue smoking during pregnancy. The parent was defined as a smoker if he smoked at least one cigarette per day. To assess smoking level we calculated the mean number cigarettes smoked per day.

### Genotyping

The genomic DNA was extracted according to a standard protocol. The gene *GSTM1– null* (GenBank accession no. X68676) and *GSTT1–null* (GenBank accession no. AP000351) genotypes were identified by the multiplex polymerase chain reaction (PCR) in peripheral blood DNA samples. The details of this method for the detection of polymorphism of *GSTT1* and *GSTM1* can be found elsewhere [35]. This method allows the detection of the presence of the genotype (at least 1 allele present: AA or Aa) or its absence (complete deletion of both alleles: aa).

Maternal blood samples were collected in vials containing EDTA and stored at a temperature of −20°C. DNA was purified from the peripheral blood using DNA purification kits (MBI “Fermentas”, Vilnius, Lithuania). DNA concentrations were quantified with a spectrophotometer (Eppendorrf BioPhotometer, 61310488, Hamburg, Germany). A PCR-based study of GSTM1 and GSTT1 polymorphism was carried out according to the method described previously [24]. The research staffs were blinded to outcome. The primers used for PCR were as follows: *GSTM1* forward5^′^-GAA CTC CCT GAA AAG CTA AAG C-3′and reverse 5^′^-GTT GGG CTC AAA TAT ACG GTG G-3^′^; *GSTT1* forward 5^′^-TTC CTT ACT GGT CCT CAC ATC TC-3^′^ and reverse 5^′^-TCA CCG GAT CAT GGC CAG CA-3^′^. As internal control, a 268-bp fragment of the human β-globin gene (GenBank accession no. U01317) was coamplified with a second set of primers (5^′^-CAA CTT CAT CCA CGT TCA CC-3^′^) and (5^′^-GAA GAG CCA AGG ACA GGT AC- 3^′^) (Biomers.net – the biopolymer factory, Germany). PCR was carried out in a final volume of 25 μl. The procedure followed for PCR was: primary denaturation at 94°C for 5 min, denaturation at 94°C for 1min, annealing at 60°C for 1 min, extension at 72°C for 1 min, 30 cycles were conducted. Final extension was at 72°C for 10 min. The PCR products were electrophoresed in 2% agarose gels and stained in ethidium bromide. The DNA bands were visualised by UV transillumination (EASY Win32, Herolab, Germany). *GSTM1* and *GSTT1* polymorphisms were coded as present (*GSTM1–1* and *GSTT1–1*) or null (*GSTM1–0* and *GSTT1–0*). To confirm the analyses we repeated genotyping for *GSTM1* and for *GSTT1* in 150 subjects. The genotyping consistency rates were 100% for both *GSTM1* and *GSTT1*.

### Statistical methods

We first examined the associations between the maternal characteristics and smoking status during pregnancy, in addition to the birthweight of the infants, by the Student’s *t*-test. We then used multiple linear-regression models to estimate the association of maternal cigarette smoking during pregnancy and the maternal genetic polymorphism in relation to birthweight of the newborn, with adjustment for major covariates. These included prepregnancy body mass index (BMI = weight/height^2^), blood pressure, parity, gestational age, education and family status. The Mantel – Haenszel test was used to test the interaction between *GSTTI* and *GSTM1* on maternal smoking. Results from these association analyses were further verified using regression models to test the associations of *GSTT1 GSTM1* and maternal smoking, including the effect from the interaction between the two genes. The subgroups were defined for maternal smoking status during pregnancy (no vs. yes) and genotypes for *GSTT1* (null vs. present) and *GSTM1* (null vs. present). We tested the gene–cigarette smoke interaction effect for birthweight reduction by adding all the product term (both 2-way and 3-way terms) in the model adjusting for potential effect modifiers. In the analyses, *beta* (β) represents the difference in mean birthweight (continuous variable) for cigarette smoking between the variant genotype after adjustment for the selected effect modifiers. Statistical significance was defined as *P* < 0.05. All statistical analyses were carried out using the SPSS software for Windows version 12.0.1.

## Results

The analysis included 543 pregnant women: 460 non-smokers and 83 continuous smokers during pregnancy. The mean number cigarettes smoked per day were 4.8. Before pregnancy smoked 140 (25.7%) study subjects, among them 42.9% continuous smokers during pregnancy and 57.1% non-smokers during pregnancy. Prevalence of passive smoking at home (husband smoking) among continuous smokers during pregnancy was 91% and among non-smokers during pregnancy it was 50.4%. A total of 95.9% women were Lithuanian and the 2 groups did not differ in ethnicity.

We also conducted analyses comparing questionnaire data and birth certificate data on various characteristics among participants and non-participants. The mean birthweight and gestational duration were similar among the two groups. These two groups did not differed by ethnic group, however, non-participating mothers were younger, less educated (did not graduate from university, 46.6% vs. 54.3%), more often smokers (smokers, 9.6% vs. 6.9%), and did have fewer prior births (no child, 64.1% vs. 45.1%), than that of participants.

We found that among 543 participant, 450 (82.9%) possessed at least one copy of the functional gene, *GSTT1–plus* genotype and the remaining 93 (17.1%) had the *GSTT1–null* genotype. The *GSTM1* gene among 543 study subjects, 293 (54%) possessed at least one copy of the functional gene, GSTM1–*plus* genotype and the remaining 250 (46.0%) had the *GSTM1–null* genotype. The carriers of the double-null genotypes comprised 8.7% of the total population studied. For the *GSTM1* and *GSTT1* polymorphisms, we were unable to determine whether they were in Hardy-Weinberg equilibrium because heterozygous individuals could not be distinguished from homozygous wild type.

Maternal characteristics with reference to the tobacco smoke exposure status are presented in Table 1. The nonexposed and exposed groups were similar in terms of the maternal prepregnancy BMI, hypertension, perceived stress, history of miscarriage, parity, sex of infant, and ethnic group, whereas the two groups differed with reference to maternal age, education and family status (*P* < 0.001). The mean birthweight of the infants was 3399.0 g for the nonexposed group and that for the exposed group was 3284.1 g, but there was no statistically significant difference (*P* = 0.132). The mean gestational age was 38.9 weeks for the both groups. In terms of the frequency of the *GSTM1–null* genotype, women in the group exposed to tobacco smoke and the group nonexposed were similar (41.0% and 47.0%, *P* = 0.340), whereas the *GSTT1–null* genotype was found in 26.5% of the smokers and in 15.4% of the non-smokers (*P* = 0.018).

**Table 1:** Characteristics of pregnant women and their newborn infants according to exposure to cigarette smoke during pregnancy

**Maternal characteristics continuous and binary**	**Maternal smoking status during pregnancy**		
	**Non-smoking (n=460)**	**Smoking (n=83)**	***P*-value**
Maternal age, mean (SD), years	29.0 (5.0)	26.4 (5.7)	< 0.001
Maternal height, mean (SD), cm	167.6 (5.7)	167.3 (7.2)	0.703
Maternal weight, mean (SD), kg	75.3 (12.5)	77.4 (15.2)	0.250
BMI, mean (SD)	26.8 (4.3)	27.6 (4.8)	0.136
Gestational age, mean (SD), weeks	38.9 (2.2)	38.9 (2.2)	0.979
Birthweight, mean (SD), g	3399.0 (639.2)	3284.1 (632.5)	0.132
Birth length, mean (SD), cm	51.3 (3.1)	50.8 (3.5)	0.150
Infant sex, n (%)			
male	245 (53.3)	47 (56.6)	0.737
female	213 (46.3)	36 (43.4)	
Education, n (%)			
university, college	437 (95.0)	53 (63.9)	<0.001
≤ 12 years	23 (5.0)	30 (36.1)	
Marital status, n (%)			
married	377 (82.0)	40 (48.2)	<0.001
not married	83 (18.0)	43 (51.8)	
Parity, n (%)			
1^st^	217 (47.2)	38 (45.8)	0.905
2^nd^ and more	243 (52.8)	45 (54.2)	
Miscarriage, n (%)			
no prior	369 (80.2)	70 (84.3)	0.450
yes	91 (19.8)	13 (15.7)	
Blood pressure, n (%)			
< 120/80 mm/Hg	324 (70.4)	56 (67.5)	0.604
> 120/80 mm/Hg	136 (29.6)	27 (32.5)	
Stress, n (%)			
no	379 (82.4)	64 (77.1)	0.281
yes	81 (17.6)	19 (22.9)	
Ethnic group, n (%)			
Lithuanian	441 (95.9)	80 (96.4)	0.571
other	19 (4.1)	3 (3.6)	
*Passive smoking*			
*Yes*	235 (50.4)	75 (91.0)	<0.001
*No*	225 (49.6)	8 (9.0)	
*GSTT1*, n (%)			
present	389 (84.6)	61 (73.5)	0.018
null	71 (15.4)	22 (26.5)	
*GSTM1*, n (%)			
present	244 (53.0)	49 (59.0)	0.340
null	216 (47.0)	34 (41.0)	

SD standard deviation of the variability of individual observations.

Table 2 presents the influence of maternal characteristics on the birthweight of the infants as the difference in mean birthweight in relation to the maternal characteristics listed in each row.

**Table 2:** **Influence of maternal characteristics on infant’s birthweight assessed by the crude and adjusted coefficient ß in linear regression (n=539**^**†**^**)**

**Maternal characteristics continuous and binary**	**ß crude**	**SE**	***P*-value**	ß^*^adjusted	**SE**	***P*-value**
BMI^**^	42.7	6.0	<0.001			
Gestational age, week^**^	191.0	9.2	<0.001			
Age < 20 or > 30 years	−34.2	56.7	0.547	−11.43	41.3	0.783
Low education, ≤ 12 years	−279.7	91.7	0.002	−155.3	67.2	0.021
Not married	−148.9	64.7	0.022	−87.1	47.2	0.066
Parity 2nd and more	49.5	55.0	0.368	90.8	39.9	0.023
Miscarriage	15.2	69.7	0.828	4.0	50.8	0.937
Previous preterm	−400.1	135.2	0.003	82.5	100.0	0.409
Maternal stress	−94.7	70.7	0.181	5.4	51.6	0.916
Blood pressure > 120/80 mm/Hg	189.4	76.6	0.014	87.5	59.0	0.139
Maternal smoking	−114.9	76.1	0.132	−137.0	55.2	0.013
Parental smoking	−86.9	55.0	0.115	−93.3	40.1	0.020
*GSTT1* null vs present	−104.1	72.7	0.153	−72.7	52.7	0.170
*GSTM1* null vs present	−25.3	55.1	0.646	−26.0	40.0	0.516
*GSTT1* null & smoking	−207.0	138.9	0.137	−211.8	100.8	0.036
*GSTM1* null & smoking	−60.4	113.3	0.594	−150.2	82.4	0.069
*GSTT1* &*GSTM1 null* and smoking	−294.0	194.4	0.131	−340.4	141.2	0.016

ß represent the difference in mean birthweight for maternal characteristics in each row.

ß^*^adjusted for body mass index, gestational age and loss outlier.

^**^Continuous variable.

SE standard error of the difference between the means.

^†^ Excluded outliers.

The characteristics that positively affected the crude mean birthweight were increased BMI, hypertension and gestational age. Low levels of education, not married status and previous preterm were associated with reduction in the mean birthweight. In terms of the *GSTM1–* and *GSTT1–* genotype frequencies, there was no significant influence on the crude birthweight of infants. After adjustment for the BMI, gestational age and loss outliers, the maternal characteristics that affected the reduction in birthweight were as follows: low education levels (−155.3 g), maternal smoking during pregnancy (−137.0 g), parental smoking (−93.3 g) and *GSTT1–null* genotype in smokers (−211.8 g, *P* = 0.036). When both *GSTT1–* and *GSTM1–null* genotypes were considered, continuous maternal smoking during pregnancy was associated with a mean reduction of 340.4 g (*P* = 0.016) in birthweight of infants.

Table 3 presents the crude and adjusted combined associations of continuous maternal smoking during pregnancy and maternal *GSTT1* and *GSTM1* genotypes with reference to infant birthweight, where β represents the difference in mean birthweight between each subgroup and the reference group.

**Table 3:** **Associations between maternal smoking during pregnancy and infant birthweight by maternal *GSTT1 *and *GSTM1 *genotype assessed by the crude and adjusted coefficient ß in linear regression**

**Genotype**	**Smoking status during pregnancy**	**Birthweight, g**	**Birthweight, g β**^**+**^**crude (SE) P**	**Birthweight, g β**^**++**^**adjusted (SE) P**	**Birthweight, g β**^**++**^**adjusted (SE) P**
Total sample	Non-smoking (n = 456)	3390.3	Referent	Referent	Referent
	Smoking (n = 83)	3284.1	−86.5 (57.5) 0.066	−83.4 (57.1) 0.073	−83.4 (57.1) 0.073
***GSTT1***					
Present	Non-smoking (n = 385)	3401.4	Referent	Referent	Referent
Present	Smoking (n = 61)	3320.7	−70.4 (65.7) 0.143	−72.3 (65.1) 0.134	−38.8 (57.6) 0.250
Null	Non-smoking (n = 71)	3330.0	Referent	Referent	−22.6 (57.6) 0.345
Null	Smoking (n = 22)	3182.9	−115.9 (129) 0.186	−123.7 (131) 0.175	−162.9 (93.0) 0.041
*Interaction: smoking × *GSTT1-null*			−111.9 (123.1) 0.182	−96.8 (122.5) 0.215	
***GSTM1***					
Present	Non-smoking (n = 242)	3413.6	Referent	Referent	Referent
Present	Smoking (n = 49)	3255.9	−87.2 (73.9) 0.119	−85.1 (74.2) 0.126	−58.8 (66.1) 0.187
Null	Non-smoking (n = 214)	3363.9	Referent	Referent	−32.4 (41.2) 0.216
Null	Smoking (n = 34)	3324.8	−97.1 (92.0) 0.146	−100.6 (90.2) 0.133	−118.7 (79.6) 0.069
*Interaction: smoking ×*GSTM1-null*			−17.0 (106.1) 0.437	−26.6 (105.6) 0.400	
***GSTT1 & GSTM1***					
Present	Non-smoking n = 207)	3429.4	Referent	Referent	Referent
Present	Smoking (n = 38)	3251.2	−137.9 (82.3) 0.048	−135.5 (82.6) 0.051	−84.7 (71.2) 0.118
Null	Non-smoking (n = 36)	3339.7	Referent	Referent	10.1 (76.1) 0.447
Null	Smoking (n = 11)	3093.5	−318.0 (198) 0.058	−320.8 (203) 0.061	−311.2 (128) 0.008
*Interaction: smoking ×*GSTT1–null* ×*GSTM1–null*			- 240.3 (164) 0.072	−234.5 (164.3) 0.078	

ß represent the difference in mean birth weight for cigarette smoking between the variant genotype.

+ ß crude.

++ ß after adjustment for the covariates: gestational age, body mass index, education, family status, parity and blood pressure.

*Test of interaction: a P value is presented for testing the null hypothesis, ß = 0 in multiple linear regression models for the product term, smoking x genotypes.

After complete adjustment for gestational age, BMI, education, family status, parity and hypertension, the reduction in birthweight (analysed as a continuous variable) for continuous smokers was 83.4 g (*P* = 0.073). Among non-smoking mothers, the *GSTT1–null* genotype alone did not confer a significant adverse effect on birthweight (−22.6 g, *P* = 0.345). The findings suggested a birthweight reduction among low-level smoking mothers with the *GSTT1–null* genotype (−162.9 g, *P* = 0.041). Maternal smoking was associated with a mean reduction of 58.8 g in birthweight for the GSTM1*–plus* and 118.7 g, *P* = 0.069 for the *GSTM1–null* genotypes; nevertheless, there was no statistically significant difference. When a combination of these genotypes was considered, a modifying effect was revealed and birthweight was significantly lower for infants of smoking women carriers of the double-null genotypes (−311.2 g; *P* = 0.008). The interaction effect of maternal smoking, *GSTM1* and *GSTT1* genotypes was marginally significant on birthweight (−234.5 g; *P* = 0.078).

## Discussion

In this molecular epidemiological study on maternal cigarette smoking and genetic determinants of xenobiotic metabolism, we found some evidence that the effects of maternal smoking on infant birthweight were modified by the maternal *GSTT1* and *GSTM1* genotypes. This study shows that even light maternal smoking (mean: 4.8 cigarettes/day) has an increased risk for infant birthweight reduction among genetically susceptible woman. Smokers with the variant *GSTT1–null* genotype had babies with lower mean birthweight (162.9 g) than non-smokers with the same genotype (P = 0.041), while smokers with the variant *GSTM1–null* genotype had babies with lower mean birthweight (118.7 g) than non-smokers with the same genotype (P = 0.069). We also found a gene-gene interaction among smokers. A combination of the *GSTM1–null* and the *GSTT1–null* genotypes has been found to exacerbate the effect of maternal exposure to tobacco-smoking on the birthweight of infants more than the presence of either genotype alone: −311.2 g, P = 0.008 in smokers vs. 10.1 g, P = 0.447 in non-smokers. An interaction effect of maternal smoking, *GSTM1* and *GSTT1* genotypes was marginally significant on birthweight (−234.5 g, *P* = 0.078). All associations were assessed after a number of relevant covariates were statistically controlled. These data and previous studies reported findings [18] suggest that the observed reductions in infants’ birthweight from this sample could be related to the main effects of prenatal exposure to tobacco.

Consistent with earlier studies, we found that maternal cigarette-smoking reduced the birthweight of infants [8,36] and that infant birthweight may vary in relation to gestational age, BMI, parity, and other variables of the population considered in the corresponding study [5,37]. Women reporting three or more stressful life events were significantly more likely to have a low birthweight infant after controlling for smoking and other socio-demographic covariates [38].

Findings of this study provide additional data supporting the conclusion that light maternal smoking during pregnancy may lead to reduced birthweight in infants. Our results corroborate the results of other studies that identification of the group of susceptible subjects should be based on both environmental exposure and gene polymorphism and that the individual differences in metabolic activation and detoxification of xenobiotics partly depends on the genetic polymorphisms associated with the GST enzymes [13-15,18,24,34]. When the *GSTT1* genotype is considered in smoking pregnant women, the three different studies estimated reduction in birthweight among the *GSTT1–plus* and *GSTT1–null* groups was as follows: 43 g (P = 0.48) [14], 222 g (*P* < 0.05) [15], and 642 g (*P* < 0.001) [13]. When the *GSTM1* genotype is considered, the estimated reduction in birthweight between *GSTM1–plus* and *GSTM1–null* groups is 171 g (*P* = 0.04) [14] and 222 g (*P* < 0.05), respectively [15]. The effects on the reduction of birthweight are not observed among women with *GSTM1–null* or *GSTT1–null* genotypes who had never smoked and the data have been adjusted to the main confounding factors.

A combination of the *GSTM1–null* and the *GSTT1–null* genotypes has been found to exacerbate the effect of maternal exposure to environmental tobacco-smoking on the birthweight of infants more than the presence of either genotype alone. Our previous publicised study have shown that when both *GSTM1* and *GSTT1* genotypes were considered, the greater increase in low birth weight and intra-uterine growth restriction risk was found among smoking mothers with the *GSTM1* genotype absent, OR 3.31 [95% CI 0.60, 18.4] and OR 2.47 [95% CI 0.31, 13.1], correspondingly [34].

We can postulate that the significant differences between the publicised studies, which are devoted to the effects of tobacco-smoke exposure on birthweight, could be attributed to the diverse ethnic composition of the populations considered in the studies, resulting in different distributions of the *GST* allelic frequency and different levels of cigarette-smoke exposure, because dose–response gradients in relation to the number of cigarettes smoked do exist [19]. Furthermore, these results may be affected by the residual uncontrolled confounding variables, such as prepregnancy BMI, hypertension, stress level, gestational age and others, which are negatively or positively associated with birthweight.

The main factors influencing birth-weight reduction are gestational age and the organism’s response to toxicity from environmental xenobiotics, such as tobacco PAHs. Tobacco smoke toxins impair placental vasculature function and subsequent transplacental transport of oxygen and nutrients, and may lead to changes in vascular resistance [39,40]. Reduction in blood flow increase apoptosis and it is possible that this could be one of the mechanisms playing a role in the growth restriction [41].

Several recent studies have investigating how genetic susceptibility modulates risk of adverse pregnancy outcomes from environmental exposures such as cigarette smoke. Toxic chemicals could disturb foetal and placental cellular regulation via elevated DNA adducts and DNA damage [42]. Oxidative damage to placental DNA and increased levels of 8-oxodG in placental DNA can result in intrauterine growth restriction and low birthweight [43]. Maternal tobacco smoke exposure at an epigenome-wide level is associated with placental gene expression and DNA differential methylation and smoking-mediated birthweight reduction [44].

It is likely that smoking mothers with high-risk genotypes may have higher levels of PAH-DNA adducts and DNA strand breakage due to the increased activity of enzymes that metabolize cigarette toxins (e.g., *CYP1A1* Aa and aa) and lower or absent activity of enzymes that detoxify these compounds (e.g. *GSTT1–null*, *GSTM1–null* genotypes) [45]. Moreover, such gene–smoking interactions may exert their synergistic effects on birthweight through maternal and foetal inflammatory responses and immune responses [46]. As reported by some authors, maternal exposure to tobacco smoke induces oxidative stress. Furthermore, maternal genetic polymorphisms related to *GSTM1* and *GSTT1* may modify the oxidative stress caused by maternal exposure to tobacco smoke [47].

We have investigated the genetic effects and the gene-environment interaction by controlling for major confounding variables. This study has the advantage of being the first to show that even light maternal smoking, in association with double-null *GSTT1* and *GSTM1* genotypes, might significantly decrease the infant birthweight. In this study, we estimated that the percentage of *GSTT1–null* genotype was 17.2% and that of *GSTM1* was 46.0%. The carriers of double-null genotypes were 8.7% of the total population studied.

When the results of this study are interpreted, a few conditions should be considered. This is a low-risk population with low-level tobacco smoke exposure (4.8 cig./day) and low prevalence of *GSTT1–null* genotype; these factors may limit the extrapolation of these results to other populations. One of the limitations of the study is the relatively small sample size with the *GSTT1–null* genotype. The evaluation of exposure to tobacco smoke was indirect; we used self-reported information on smoking during pregnancy, and thus the possibility of bias in both reporting and exposure classification exists. We also examined phase-II metabolic genes without study genes expressed in phase-I. However, in this study, we controlled for the main variables that might confound the association between maternal smoking, genetic polymorphism and birthweight; therefore, the residual confounding of the results by smoking is expected to be small. Despite these limitations, findings of this study provide additional data supporting the conclusion that maternal smoking during pregnancy may lead to reduced birth weight in newborns.

## Conclusion

The study shows the modifying effect of the *GSTT1* and *GSTM1* genotypes on birthweight among smoking women and presents evidence that carriers of the null genotypes should be treated as an increased susceptibility group for infant birthweight decrease. Our findings provide additional insight into the biological determinants of response to environmental exposure based on the combination of genes and individual characteristics. Genotyping for the *GSTT1* and *GSTM1* polymorphisms, simple and inexpensive assays, could be suitable biomarkers identifying genetically susceptible pregnant women. These risk stratification markers could provide a valuable approach to estimate the "causal" effects of risk behaviours with genetic-predisposing factors (such as smoking) and could lead to targeted smoking cessation interventions during pregnancy as prevention for infants with low birthweight. The *GSTT1–null* genotype, either presents only one or both with *GSTM1–null* genotype in a single subject, may have a modifying effect on birthweight among smoking women even though their smoking is low level. Our data also show that identification of a susceptible-subject group should be based on both environmental exposure and gene polymorphism.

## Competing interest

We confirm that all authors have no actual or potential competing interests regarding the submitted article and the nature of those interests.

## Authors’ contributions

AD was involved in primary data collection as well as the coding and analysis of data, and the preparation of the manuscript. RG was involved in the conceptualization of the research, the preparation of the manuscript. AP contributed to genetic analysis. RD contributed to the development of the survey instrument, contributed to data acquisition. MJN contributed to qualitative data analysis and contributed to revisions of the manuscript. All authors read and approved the final manuscript.

## Pre-publication history

The pre-publication history for this paper can be accessed here:

http://www.biomedcentral.com/1471-2393/12/161/prepub
